# Microfluidic Synthesis of Iron Oxide Nanoparticles

**DOI:** 10.3390/nano10112113

**Published:** 2020-10-23

**Authors:** Matthew James, Richard A Revia, Zachary Stephen, Miqin Zhang

**Affiliations:** 1 Department of Materials Science and Engineering, University of Washington, Seattle, WA 98105, USA; mattj71@uw.edu (M.J.); rrevia@uw.edu (R.A.R.); zrs420@uw.edu (Z.S.); 2 Department of Neurological Surgery, University of Washington, Seattle, WA 98105, USA

**Keywords:** iron oxide nanoparticle, microfluidic, coprecipitation, scale up, manufacturing, materials

## Abstract

Research efforts into the production and application of iron oxide nanoparticles (IONPs) in recent decades have shown IONPs to be promising for a range of biomedical applications. Many synthesis techniques have been developed to produce high-quality IONPs that are safe for in vivo environments while also being able to perform useful biological functions. Among them, coprecipitation is the most commonly used method but has several limitations such as polydisperse IONPs, long synthesis times, and batch-to-batch variations. Recent efforts at addressing these limitations have led to the development of microfluidic devices that can make IONPs of much-improved quality. Here, we review recent advances in the development of microfluidic devices for the synthesis of IONPs by coprecipitation. We discuss the main architectures used in microfluidic device design and highlight the most prominent manufacturing methods and materials used to construct these microfluidic devices. Finally, we discuss the benefits that microfluidics can offer to the coprecipitation synthesis process including the ability to better control various synthesis parameters and produce IONPs with high production rates.

## 1. Introduction

IONPs have gained much attention due to their versatile applications in nanomedicine as diagnostic and therapeutic agents in the fight against human disease. IONPs can be used as contrast agents in magnetic resonance imaging or fluorescence imaging [1,2,3,4], small-molecule drug carriers in drug delivery [5,6], transfection vectors in gene therapy [7,8], enhancers for magnetic hyperthermia [9,10], and various roles in other applications [11]. IONPs have proven to be nontoxic and biodegradable [12,13,14]. Multiple IONP formulations have received approval from the United States Food and Drug Administration (FDA) for medical applications, and one of the most recent examples is Feraheme (ferumoxytol) for remediation of iron deficiency anemia [15,16].

There are a number of methods for synthesis of IONPs, including thermal decomposition, microwave-assisted growth, laser-ablation, and coprecipitation, each with its own advantages and disadvantages. The thermal decomposition technique yields highly monodispersed nanoparticles through the use of processing high temperatures and harsh chemicals [17]. Although the IONP size is well controlled in the thermal decomposition method, the technique is energy, material, and time intensive, and has a very low production rate (0.002 g/h) [17,18]. The microwave-assisted synthesis yields a large production rate of IONPs (0.88 g/h) [19], but the equipment required for this technique is highly specialized, expensive, difficult to manufacture, and must be able to handle high pressures [20]. The laser-ablation technique can also produce IONPs upon laser exposure for short amounts of time, but IONPs thus created are often polydisperse, too large in size for use in biological environments, and have long down times in between reaction steps [20]. Further, the laser-ablation technique has a low production rate of IONPs (~0.013 g/h) [21].

**Figure 1:**
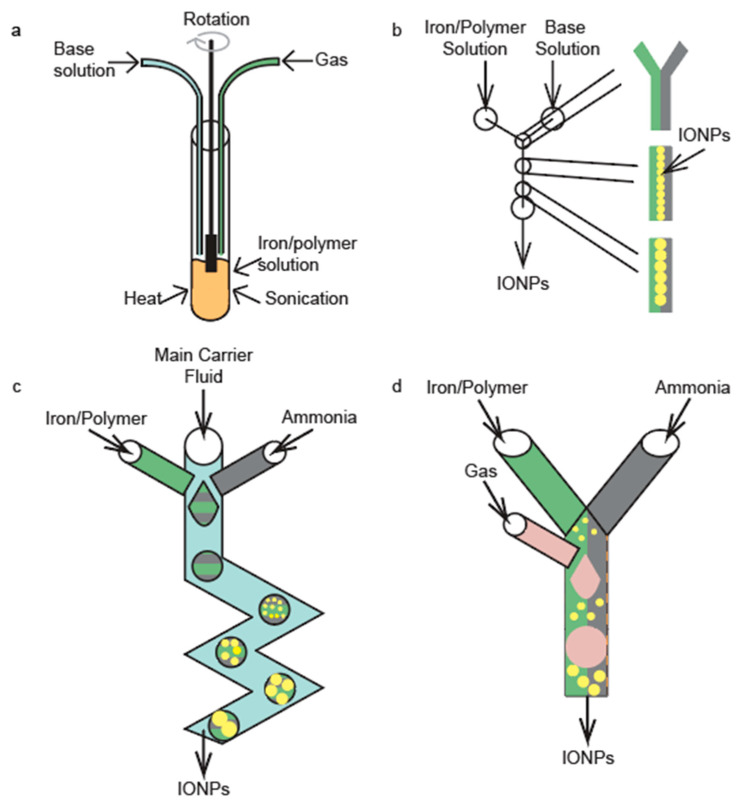
Illustrations of a (**a**) standard coprecipitation synthesis, (**b**) continuous-flow reactor, (**c**) drop-wise flow reactor, and (**d**) gas-segmented flow reactor.

One of the most common synthesis techniques for producing IONPs is the coprecipitation of ferric and ferrous iron salts in basic aqueous solution. The standard coprecipitation procedure is illustrated in Figure 1a. The technique is fairly simple and requires little effort in terms of set up. In essence, a solution of dissolved iron is mixed with a polymer solution or surfactant in a container, followed by the addition of a basic solution to raise the pH. In addition, heating, sonication, and/or mechanical stirring may be applied to ensure even growth of IONPs throughout the solution [22,23,24].

The precipitation of iron-oxide spheres from a solution of iron salts relies on iron oxide’s pH-dependent solubility in the solution [25]. The iron salt solution becomes super saturated at a pH above ~10, at which point iron oxide begins to nucleate and subsequently grow in the solution [26]. The uniformity of the nucleation and growth can be improved by mechanical stirring and reducing the reaction volume [27]. Thus, one of the main drawbacks of the standard coprecipitation synthesis method is the difficulty to scale up for mass production. The larger the reaction volume, the more difficult to achieve homogeneity [27]. Additionally, gas, such as nitrogen, can be blown over the sample to help maintain the pH and can also prevent oxidation of the materials [28]. Due to the lack of control over mixing and other parameters, traditional coprecipitation techniques may yield IONPs with a broad size distribution, and batch-to-batch inconsistency in IONP size, crystallinity, morphology, and other physicochemical properties [29].

To overcome these drawbacks and produce high-quality IONPs in large quantity, a recent effort has been made on developing microfluidic devices to make IONPs. A microfluidic device is made of any series of interconnected micron-scale channels etched into or formed from a variety of materials. Specifically, microfluidic devices are generally defined as being able to manipulate fluid volumes between nano- and microliters [30]. Due to the small scale of the channels composing microfluidic devices, reaction environments can be precisely controlled so that homogeneous reaction volumes are achieved within the channels [24,25,26]. The micron-scale size of the channels in a microfluidic device dictates that the Reynolds number of the fluids flowing in the channels is so low that laminar flow will be observed [31,32]. Laminar flow allows for excellent control over the kinetic parameters of the two solutions to be mixed during the coprecipitation of IONPs (i.e., the iron salt solution and the base solution) [33]. As a result of this laminar flow, the nucleation and growth of IONPs is generally diffusion controlled in microfluidic devices [31]. Furthermore, these small-scale channels provide large surface area-to-volume ratios, which enhances the homogeneity of the solution and in turn increases the heat and mass transport in the solution [33].

Microfluidics can be used to aid in the coprecipitation of IONPs by taking an iron solution with dissolved polymer or surfactant, a base solution, and any other solutions or gasses necessary, and flowing them through the microfluidic device [33]. There are generally two types of microfluidic devices: tubular and chip reactors [34]. The main difference between the two are the channel geometries; namely, tubular reactors often have circular cross-section channels, whereas chip reactors have channels with rectangular cross-sections. There are multiple different architectures that can be achieved within a microfluidic device, each with their own merits and limitations.

As an important and emerging technology, the principle and current status of the microfluidic synthesis of IONPs has been seldom reviewed [35]. This review aims to provide a current overview of the microfluidic coprecipitation of IONPs. First, we discuss the most prominent microfluidic architectures for production of IONPs, including tubular microreactors in both continuous-flow and drop-wise schemas, and chip-based reactors. We then review the most commonly used manufacturing methods and materials for the creation of microfluidic devices for IONP production; we include a discussion of the important design choices in device manufacturing methods and material selection. Finally, we discuss the unique benefits that the microfluidic devices can bring about such as the ability to better control key synthesis parameters for improved properties of IONPs and to enable large-scale production.

## 2. Microfluidic Architectures for Coprecipitation of IONPs

There are a multitude of microfluidic device architectures employed in the coprecipitation of IONPs. These structures all take the advantage of the small scale of the channels to produce high-quality, monodispersed IONPs that are consistent with respect to their physicochemical properties between synthesis batches. The first major advantage of the small channels is that they limit the reaction volume where nucleation and growth of IONPs occurs, thereby improving the homogeneity of the resulting particles. Secondly, at these small scales, laminar flow is achieved, meaning the solutions flowing through these channels have no mixing. This leads to a coprecipitation synthesis of IONPs that is heavily diffusion dependent. The diffusion of iron and base towards the center of the microfluidic device’s channel is what drives the nucleation and growth of the IONPs. Schematics of the standard coprecipitation synthesis approach and the three most commonly used microfluidic architectures are shown in Figure 1.

### 2.1. Continuous-Flow Reactors

Continuous-flow reactors have two inlets—one for an iron/polymer solution and the other for a base solution (Figure 1b). As the iron and base solutions are pumped through the microfluidic device, IONPs begin to form at the interface between the iron-polymer solution and the base solution, as this is the only point in the channel where iron is present in the solution and the pH is high enough to facilitate nucleation. The IONPs are then able to grow via diffusion of free iron and polymer in the iron-polymer solution towards the laminar interface. Laminar flow regimes are of paramount importance in continuous-flow reactors. This type of microfluidic devices must maintain a Reynolds below 2000 in order to achieve laminar flow [36]. The four aspects of the solution that affect the Reynolds number are the fluid velocity, channel diameter, fluid density, and fluid viscosity [36]. There are two common types of continuous-flow reactors, i.e., the tubular and chip reactors. Tubular continuous reactors are defined by their circular channels and can be made in house but are widely available for purchase in the form of Y or T connectors. There is a limited availability of chip reactors for purchase and therefore they are generally fabricated in house through various fabrication techniques.

The main advantage of this technique is its ability to produce IONPs in large quantity as compared to the other two microfluidic architectures. All of these architectures may be up scaled, ran in parallel, or have some key parameters such as flow rate altered to enhance the production rate. If we assume these aspects to be the same across the continuous, drop-wise, and gas-segmented flow reactors and measure the net flow out of the microfluidic device we will see a higher net output in the continuous-flow reactor than in the other two architectures. This is due to the use of carrier fluids in drop-wise flow reactors and gas slugs in gas-segmented reactors. Another advantage of the technique is the simple design and easy manufacturing of the channel geometries. A major disadvantage of this technique is less control over the reaction environment compared to the other two architectures because the other two techniques further minimize the reaction volumes by segmenting them with liquids or gasses. Therefore, continuous-flow reactors have the lowest solution homogeneity among the three microfluidic techniques.

**Figure 2:**
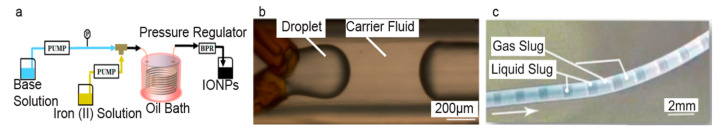
Microfluidic devices used for synthesizing IONPs demonstrating architecture types. (**a**) Continuous-flow. [37]. (**b**) Drop-wise. [29], and (**c**) gas-segmented reactors. Reproduced with permission from [38]. Copyright American Chemical Society, 2015.

An example of a continuous-flow reactor is shown in Figure 2a. In this study IONPs were synthesized with core sizes ranging from 26.5 to 34 nm [37]. The effects that various dissolved gasses would have on IONP core size were investigated, and it was found that dissolving carbon monoxide gas into the solution reduced the IONP’s core size [37]. This trend is seen because the presence of the gas can affect the chemical reactions that form the IONPs. Namely, carbon monoxide can act to cap the particles and control their shape as well as size. Additionally, a mild oxidant was added in the base solution to help promote the formation of a magnetic structure [37].

In another study with a continuous-flow reactor, IONPs were synthesized with core sizes ranging from 8 to 56 nm [39]. In this study, a base-polymer solution and an iron solution were used, and it was found that increasing the flow rate of both solutions increased the core sizes of the IONPs [39]. The synthesized IONPs were evaluated for their magnetic properties for applications in magnetic hyperthermia. It was determined that IONPs synthesized at a flow rate of 0.04 mL/s and coated with 6% Dextran from Leuconostoc were more stable and able to reach higher temperatures when compared to IONPs that were uncoated or coated with negatively charged Dextran with sodium salt [39]. It is interesting to note that in this study [39], the IONPs were not exposed to any external heat post synthesis, meaning the flow rate trend we see is a characteristic of the flow rate and not an influence of heating. Additionally, the importance of polymer type is made apparent here by the difference in effectiveness of heating.

### 2.2. Drop-Wise Flow Reactors

Drop-wise flow reactors further promote the small reaction volumes by dividing the main solution into droplets, so that the reaction homogeneity is further improved. This technique is schematically illustrated in Figure 1c. Drop-wise flow reactors generally have three inlets, two for the primary mixing solutions and one for a carrier fluid. The two inlets for the primary mixing solutions are angled into the main channel such that they are able to form droplets within the carrier fluid. As the carrier fluid is continually pumped through the channels, droplets are continuously formed and transported along the channel. Within a droplet, the advective convection drives the creation of IONPs. Both tubular and chip reactors have been developed to make IONPs by this drop-wise technique.

Drop-wise flow reactors take advantage of a microfluidic channel’s small dimensions to minimize reaction volumes and achieve advective convection [40], which leads to reaction conditions that yield uniform IONPs due to homogenous mixing within the droplets. This homogeneity may be enhanced by altering the channel geometries to promote mechanical mixing of the droplet [40]. The capillary number is a measure relating the viscous forces and surface tension forces between a liquid and gas or two immiscible liquids, and it plays a role in the effectiveness of this type of microfluidic device [40,41]. At low capillary numbers, the droplet undergoes little to no deformation, which is ideal when trying to maximize the homogeneity of the droplets [41].

A major disadvantage of this technique is that the production rate is generally lower than that achieved in continuous-flow reactors of comparable parameters aspects, such as fluid velocities, due to the segmentation of the fluid in the drop-wise flow reactors. Further, these reactors generally require more complicated geometrical designs than continuous-flow reactors to accommodate the addition of the carrier fluid and the combination of the two primary mixing solutions to form droplets. These complexities in geometry limit manufacturing methods that can be used to fabricate the devices. One of the major advantages to this technique is that, due to the carrier solution, the droplets have very low degrees of interactions with the surfaces of the channel and therefore mostly avoid any particle deposition on the channel walls, which in turn help prevent the fouling of the channels [29]. Drop-wise flow reactors also tend to have the highest level of homogeneity of the three reactors because the droplets are formed directly in the carrier solution.

IONPs with ultrasmall core sizes of 4 ± 1 nm have been produced using drop-wise flow reactors [42]. The magnetic properties of these IONPs were evaluated by measuring the magnetization as a function of magnetic field [42]. There was an absence of a hysteresis loop, indicating that the IONPs are superparamagnetic; this coupled with HRTEM data verified that the IONPs were superparamagnetic and of the spinel structure [42]. IONPs with a very narrow size distribution have also been synthesized with average core sizes of 10.5 ± 0.1 nm using a drop-wise flow reactor [29]. An example of the microfluidic device used can be seen in Figure 2b. These results indicate that drop-wise flow reactors have the potential to provide excellent control over formation of IONPs and thus can produce smaller IONPs as compared to a standard coprecipitation method [42]. Smaller IONPs provide larger surface area-to-volume ratios, and are desirable, in most of biomedical applications. Another study showed that while both the drop-wise and standard coprecipitation methods yielded similar core sizes, the size distribution of the drop-wise IONPs was better than that made with the standard coprecipitation [29]. Narrow size distributions are desirable; broad size distributions can be a hindrance to applications that require monodisperse IONPs [29]. A broad size distribution can be indicative of IONP agglomeration and solution heterogeneity.

### 2.3. Gas-Segmented Flow Reactors

Gas-segmented flow reactors are very similar to drop-wise flow reactors in that they further minimize the reaction volumes. In this technique, iron-polymer and base solutions are mixed to form the main solution; then, a gas is introduced to segment the main solution into slugs. This segmentation reduces the reaction volume of the main solution. An illustration of the principle of the device operation is shown Figure 1d. The major difference between gas-segmented flow reactors and drop-wise flow reactors is that gas-segmented reactors use gas to form and separate slugs after the main solution has been formed as opposed to droplets being formed in a liquid carrier fluid. IONPs form via diffusion-controlled coprecipitation in these schemas. The advantages of this technique are similar to those of the drop-wise flow reactor (i.e., a high-degree of IONP reaction homogeneity yields highly monodisperse IONPs); further, this technique allows access to the use of different types of gasses, which can alter the size and shape of the IONPs [38]. The main drawback of this technique is that the main solution is formed in bulk and then separated into smaller portions, which may lead to lower homogeneity in the solution when compared to the drop-wise flow reactors [43]. Additionally, these gas slugs can cause large high friction in the system, which can be minimized by coating the inner walls of the channels with a surfactant [44,45].

One study used a gas-segmented flow reactor to test the effect of different gasses on the IONP’s properties [38]. IONPs were synthesized with core sizes ranging from 23 ± 6 to 70 ± 12 nm [38]. It was also shown that the size and shape of the IONPs could be varied based on the gas used to segment the slug [38], indicating that this technique can be used to synthesize a broad range of shapes and sizes of IONPs. Specifically, hydrogen and nitrogen slugs formed cubic particles, oxygen formed spherical or rod-shaped particles, and carbon monoxide slugs formed hexagonal shaped particles [38]. Additionally, a mild oxidant was added to the reaction to help with the formation of magnetic phases [38]. An example of this device is shown in Figure 2c.

All three architectures discussed above provide multiple advantages over the standard coprecipitation method. Most importantly, these methods allow for the continuous production of IONPs [29,37]. Drop-wise flow reactors provide the highest level of homogeneity when compared to continuous-flow reactors and gas-segmented flow reactors. This means that drop-wise flor reactors have the best solution conditions to form highly monodisperse particles at the cost of production rate. While Reynolds number, capillary number, and friction play a role in each of these architectures, their individual contributions to the architectures where they are introduced play a pivotal role in preserving the homogeneity of the solution.

## 3. Fabrication of Microfluidic Devices for Production of IONPs

**Figure 3:**
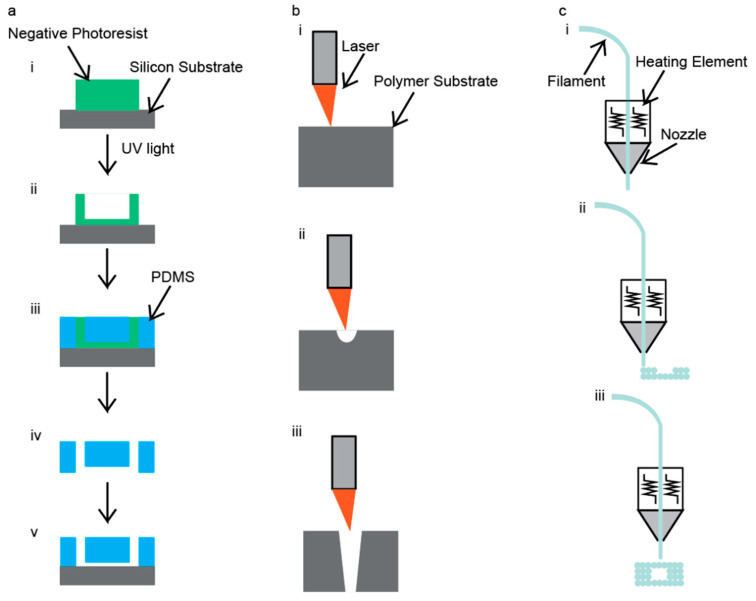
Illustration of the fabrication techniques for fabrication of IONP-producing microfluidic devices: (**a**) photolithography, (**b**) laser cutting, and (**c**) 3D printing.

Microfluidic devices can be manufactured by a variety of techniques; however, there are three main techniques that are employed: photolithography, laser cutting, and 3D printing [46]. Each of these techniques has advantages and disadvantages, therefore, specific aspects of the microfluidic device need to be considered before choosing a fabrication technique. First among these considerations is the desired accuracy and precision of the channel geometries, which can play a critical role in the synthesis of IONPs, especially if channel aspect ratios and dimensions are points of optimization in the system. In addition, surface roughness also needs to be considered. Surface roughness can play a role in contamination in the system by trapping IONPs from previous runs and then reintroducing them into a later batch. A final consideration is the complexity of the channels’ geometries, which can limit the availability of applicable fabrication techniques as well as play a role in determining the accuracy and precision of the channels. The fabrication technique determines the level of difficulty in the creation of channel geometries. Figure 3 illustrates three common techniques that have been used to fabricate microfluidic devices specifically for the coprecipitation of IONPs.

### 3.1. Photolithography

Photolithography uses ultraviolet (UV) radiation to ablate the surface of a negative photoresist layer to define a microfluidic device’s channel geometry [46]. A graphic illustration of this method is shown in Figure 3a. After the microchannels have been defined by the UV light, an inverse image of the mold is obtained using a polymer before binding to a glass substrate. The main advantage of this technique is that the chips are made with precise geometries within the channels and excellent surface smoothness is achieved [47]. The main disadvantages of this technique are that it requires a lot of time, use of toxic materials, and clean-room facilities [47]. As a result of diffraction, the lower resolution limit of this technology is 250 nm [48]. Further discussion on photolithography of microfluidic chips can be found in an article by Duarte et al. [48].

**Figure 4:**
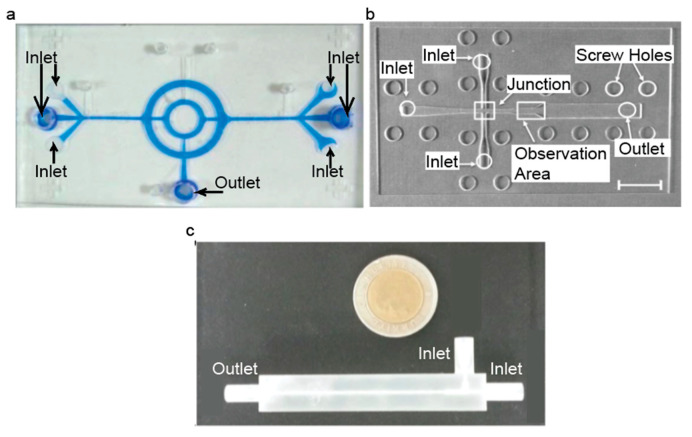
Images of microfluidic devices that are fabricated using (**a**) photolithography (Reproduced with permission from [51]. Copyright Springer Nature, 2008), (**b**) laser cutting (Reproduced with permission from [52]. Copyright Elsevier, 2014.) and (**c**) 3D printing (Reproduced from [53].)

Several studies have successfully produced IONPs using a device produced by photolithography. Polymers (PDMS and SU-8) were used to fabricate microfluidic devices used to produce IONPs with core sizes of approximately 10 nm in two separate studies [49,50]. Additionally, a PDMS and glass chip microfluidic device was fabricated using photolithography and utilized for IONP synthesis to produce NPs with core sizes ranging from 4.83 ± 1.20 to 6.69 ± 1.15 nm [51]. An image of this microfluidic device is shown in Figure 4a. It was found that unstable flow rates caused a higher density of IONPs to form in solution but caused less stable droplets to form [49], and as the flow rates saturated the droplets stabilized, but IONP density decreased [49]. For some drop-wise flow reactors, the droplets formed have larger diameters than the channel itself, and due to surface energy, these droplets tend to change back into spheres. Wells have been incorporated into microfluidic devices in an attempt to further improve the homogeneity of the samples [49]. Droplets are formed and are compressed until they reach a well, where they form into a sphere. The next droplet moves down until reaching the well, and then pushes the first droplet out. This incorporation can eliminate variations in droplet size [49], which can be useful for further improving the homogeneity of the droplets.

### 3.2. CO_2_ Laser Cutting

CO_2_ laser cutters use high-powered lasers to ablate the surfaces of materials to make cuts or channels. An example of this technique is shown in Figure 3b. Generally, these cuts and channels are made on polymer materials, such as poly(methyl methacrylate) (PMMA). This technique requires a 2D drawing to be uploaded to the laser cutter. The laser cutter then takes the 2D drawing and cuts out the microfluidic device. There are two types of actions that can be performed. The first type of action is etching, and this maneuver does not go all the way through the polymer but instead removes a few layers of the polymer off of the surface, which can be seen in Figure 3(bii). The second type of action is referred to as a cut. This maneuver goes all the way through the polymer, as shown in Figure 3(biii).

Some advantages of this technique are that it uses inexpensive and nontoxic materials and it is a facile process; yet most polymers can be used, because the chamber housing the laser cutter can be filled with nitrogen to prevent the polymers and many other materials from combusting [46]. A major disadvantage of this technique is the initial cost of purchasing the laser cutter and surface roughness in the laser-cut channels [46], which may entrap solution or IONPs from previous uses. Laser cutting is a simpler process than photolithography but yields lower channel precision. Laser type, such as femtosecond and IR lasers, can also provide distinct advantages such as generating more accurate channel geometries and shorter manufacturing times [54,55]. More details on the laser cutting of microfluidic devices can be found in two articles by Malek [54,55].

Yang et al. successfully produced a microfluidic device from laser-cutting PMMA and producing large chitosan droplets with multiple IONPs in each droplet [52]. It was found that increasing the flow rate of the iron solution while maintaining a constant carrier fluid flow rate increased the overall droplet size [52]. An example of this device is shown in Figure 4b. Uniquely, this study formed chitosan droplets that encapsulated an iron solution, and then these chitosan droplets were dripped into a base solution, which caused IONPs to form within the chitosan droplets [52]. The chitosan droplets are formed into spheres, and then as they are dropped into the base solution, they deform into droplet shapes. The base solution serves to both precipitate IONPs and cross-link the chitosan droplets [52]. They also used these chitosan droplets to study the controlled release of a drug and found that the chitosan droplets that had IONPs in them released the drug faster than the chitosan droplets with no IONPs [52]. This discrepancy provides an interesting application in being able to control how quickly and for how long the drug can be released.

### 3.3. 3D Printing

3D printers use high temperatures to extrude polymers to define a microfluidic device’s channel geometry in fusion deposition modeling. An illustration of this technique is shown in Figure 3c. Additionally, there are some 3D printers that can be used to 3D print with metals and glass, but these require even higher temperatures to operate. This technique has the unique advantage of having a fairly hands-off approach. First, the chip must be diagrammed using a computer-aided design program; the design files are then sent to the 3D printer. Then, the 3D printer heats the filament and extrudes it according to the diagram file, after which the filament cools, leaving behind the 3D-printed structure. A major advantage to this technique is the ability to create complicated channel geometries [37,38]. The main disadvantages of this technique are that it can be inaccurate due to the polymer shrinking upon cooling, and the available polymers that can be used are mostly thermoplastics [56,57]; however, some thermosets can be 3D printed [58]. Some other disadvantages of 3D printing are limited printing resolution and limited build sizes. These disadvantages can be improved upon; however, the cost of the printer will increase as these disadvantages are reduced. A more comprehensive review of 3D printing techniques of microfluidic devices can be found in an article by He et al. [59].

A 3D-printed microfluidic device has been used to synthesize IONPs with core diameters ranging from 30 to 150 nm [53]. It was shown that IONP core size could be controlled by altering the flow rates of the base and iron solutions, and they found that a flow rate ratio of 1:1 produced the most monodispersed IONPs as compared to other flow rate ratios [53]. An example of this device is shown in Figure 4c.

Manufacturing techniques have a significant influence on key aspects of the microfluidic device such as dimensional accuracy, channel size, channel complexity, and surface roughness. Photolithography is able to produce the highest level of accuracy, smallest channel sizes, and lowest surface roughness among the three manufacturing techniques. However, it is difficult to make complex channel geometries using this technique. 3D printing is able to produce mediocre levels of accuracy, larger channel sizes, and mild surface roughness, and more complex channel geometries with relative ease. Laser cutting produces microfluidic devices with the lowest level of accuracy, mediocre channel sizes, and the highest surface roughness. However, it can produce complex channel geometries with some ease. Overall, manufacturing technique has little effect on IONP size.

## 4. Materials Used in the Creation of IONP-Producing Microfluidic Devices

Synthesis-specific factors play a large role in what materials are available to use in the fabrication of microfluidic devices. First, and perhaps most importantly, chemical resistivity should be considered. For IONP synthesis, resistance to strong bases is one of the most important considerations. Additionally, cleaning of the microfluidic device also needs to be factored into the consideration for choice of material. Chemicals such as strong acids are commonly used to remove any left behind solution in the chip’s channels. Therefore, a material that has a resistance to both strong acids and strong bases should be chosen.

In addition to chemical resistance, cost is also an important factor in deciding what material to use. Certainly, for lab-scale synthesis projects, cost may not play a huge role, but when considering scaling up production or commercialization of the product, cost can become a limiting factor in the range of materials available. Furthermore, ease of manufacturing is also an important factor to consider when deciding on what material to use. Material choice can dictate what manufacturing techniques are available, and in turn can also determine how accurate design architectures will be rendered in the actualized microfluidic device. Additionally, materials are often used in tandem with one another to provide benefits of both materials and to allow the use of additional manufacturing techniques that require the use of diverse materials.

### 4.1. Polymers

Polymers are perhaps the most common materials used for the fabrication of microfluidic devices [46]. Polymers that are routinely used include polytetrafluoroethylene (PTFE), PMMA, and PDMS [46]. Polymers have a wide range of chemical resistances, are low cost and moderately easy to machine. There are multiple manufacturing techniques that use polymers, including photolithography, 3D printing, laser cutting, and hand techniques [46]. PTFE has the broadest range of chemical resistances, but a moderate Young’s modulus [60]. PDMS has moderate chemical resistivity and the lowest Young’s modulus [61]. PMMA has the narrowest range of chemical resistance and the highest Young’s modulus [60]. All these polymers are hydrophobic; however, if hydrophilic channels are desired there are various treatments that can be performed on the microfluidic device that can render the channels hydrophilic [62,63]. Additionally, increasing the molecular weight of these polymers enhances the chemical resistivity of the polymer but makes machining more difficult.

As mentioned earlier, providing the highest level of homogeneity for the reactant solution is extremely important when synthesizing IONPs. Due to the nature of polymers and some of the fabrication techniques that use polymers, surface roughness of the channels can distort the fluid flowing through them. To minimize this roughness, a variety of alterations can be made to both the manufacturing technique and to the channel itself. Polymers that have undergone laser cutting often experience high levels of surface roughness, which can be minimized by altering key parameters such as laser strength or number of passes [52]. For both laser cutting and 3D printing, the channels can be coated with a compatible coating or etched to reduce the surface roughness [64,65].

**Figure 5:**
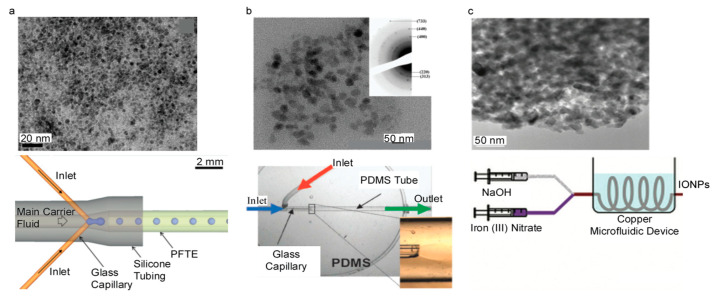
TEM images of IONPs and the respective schematics of microfluidic devices mainly made from (**a**) PFTE (Reproduced with permission from [68]. Copyright Royal Society of Chemistry, 2012), (**b**) glass (Reproduced with permission from [69]. Copyright Royal Society of Chemistry, 2008) and (**c**) copper (Reproduced with permission from [70]. Copyright Walter de Gruyter, 2011).

A number of studies have been performed on microfluidic devices manufactured using PDMS [66], PTFE [67], and PMMA [52]. IONPs produced in these microfluidic devices exhibited core sizes of 35 nm for the PDMS-based device and a core size range of 12.3 to 24.6 nm in the PTFE-based device [66,67]. Another study used PTFE, silicone, and glass capillaries to manufacture a tubular microfluidic device with channel diameters of 400 µm for the glass capillaries and 820 µm for the PTFE and 3600 µm for the silicone tubing [68]. IONPs were synthesized with core sizes of 3.6 ± 0.8 nm [68]. An illustration of the microfluidic device and a TEM image of the synthesized IONPs are shown in Figure 5a. Characterization techniques have been incorporated into the process to provide instant feedback on particle characteristics. NMR has been incorporated into a microfluidic system to provide instant feedback on particle magnetic properties [67]. With this, it is easy to visualize integrating a dynamic light scattering size analyzer into a microfluidic system to obtain instant feedback on hydrodynamic size. More complicated techniques, such as SEM or TEM, may be more difficult to integrate due to the nature of the characterization technique. However, it would be feasible to incorporate these techniques for commercialization to ensure quality.

### 4.2. Glass

Glass is another common material used in the fabrication of microfluidic devices. Glass has an excellent chemical resistivity, fairly low cost, and high ease of manufacturing [71]. There are multiple manufacturing methods that use glass to make microfluidic devices, including photolithography and hand techniques. Glass capillaries are most commonly used in microfluidic devices and can be easily incorporated into other materials. Glass capillaries tend to have low surface roughness as well, further minimizing any turbulent affect. Additionally, glass tends to be hydrophilic, which is desirable for moving a liquid through a channel.

Glass capillaries and PDMS tubing were used to manufacture a tubular microfluidic device with channel diameters of 150 and 1700 µm, respectively [69]. IONPs were synthesized with a core sizes of ~7 nm [69]. An illustration of this technique and a TEM image of the synthesized IONPs are shown in Figure 5b. It was found that lowering the stability of the solution streams led to a decrease in IONP concentration because of an increase in surface area between the two solutions [69]. Another study used a commercially available, all glass chip microfluidic device with channel dimensions of 300 µm wide and 60 µm deep, and the synthesized IONPs had a core size of 3.6 ± 1.0 nm [72]. In systems that flow one solution inside of another solution, optimization of the flow rates, also referred to as flow focusing, can enhance the homogeneity of the solution. At nonoptimal flow rates, the solutions can form turbulence in the channels. It has been theoretically predicted and experimentally confirmed in one system [69], that the exterior to interior flow rate ratio was optimal at approximately a value of 4 [69]. This is a fairly uncommon technique in the microfluidic coprecipitation of IONPs. However, it maximizes the interfacial area of the two solutions that contact each other. This greater interface aids in the diffusion of species and speed up the reaction process.

### 4.3. Metal

Metal is another material used in the fabrication of microfluidic devices. Metals have good chemical resistivity, are low cost, and easy to machine [73]. Metal microfluidic devices are most commonly machined to meet the specifications of the microfluidic device. Metals can withstand high temperatures better than polymers and are easier to machine than glass. Aluminum, copper, and iron are common metals used in microfluidic devices; however, these metals are usually alloyed with other metals to fine-tune their chemical resistance [33].

Metals have high heat transfer coefficients, and as such, small changes in temperature near the device can cause local temperature fluctuations in the microfluidic channels [74]. These fluctuations can cause inconsistencies in IONP formation. This drawback can be mitigated in a number of ways, but most generally the device can be submerged in a water or oil bath to help minimize temperature fluctuations.

A tubular microfluidic device has been fabricated out of copper with an 800 µm wide diameter [70]. IONPs were synthesized with core sizes ranging from 6.3 to 9.8 nm [70]. An illustration of this device is shown in Figure 5c. These IONPs were synthesized with a flow ratio of iron solution to base solution of 1:1 and a range of flow rates were tested spanning from 20 to 60 mL/h [70]. The flow rate also directly controlled how long the IONPs were exposed to elevated temperatures in the water bath. It was found that the particle size was at a maximum of approximately 40 mL/h. Furthermore, when comparing this system to a commercially available system at similar flow rates, but at different residence times due to differing channel geometries [70], it was found that no correlation between flow rate and particle size in the commercial system was observed but on average, the system made of copper produced smaller IONPs for similar flow rates as compared to the commercial system [70]. Exposure of IONPs to elevated temperatures plays a large part in the kinetics of the IONP formation. The more energy driven into the system, the more the particles can grow. This increase in energy also allows for a greater chance for particles to grow nonuniformly, which can cause a larger particle size distribution.

Material choice significantly impacts some key aspects of the fabricated microfluidic device including ease of manufacturing, chemical resistance, and cost. Depending on the design constraints dictated by the desired channel geometries, certain materials may need to be avoided so that a material paired with its manufacturing techniques may meet the geometry requirements. Importantly, the chemical resistance of a material determines what types of compounds may safely interface with the channels of the microfluidic device. Material choice has little effect on IONP size but does impact the choice of reaction parameters.

## 5. Experimental Design Parameters and Their Control over IONP Synthesis

As discussed in the introduction, the coprecipitation synthesis of IONPs typically involves two solutions: (1) a mixture of ferric and ferrous salts dissolved in water along with a polymer and (2) a basic solution (e.g., NaOH and NH_4_OH). Upon combining these two solutions, nuclei of iron oxide form and grow until they are encapsulated in polymer, resulting in IONPs. Given this description of the coprecipitation technique, a few important synthesis parameters are elucidated: the ratio of iron solution to base solution, concentration and type of polymer, ratio of ferric iron to ferrous iron, strength of base, and temperature. All of these parameters may be effectively controlled in a microfluidic device, and this is a main benefit for use of microfluidic devices in the synthesis of IONPs; controlling these reaction parameters has a direct impact on the properties of the resulting particles.

### 5.1. The Effect of Coprecipitation Synthesis Parameters

A few of the synthesis conditions mentioned above must be determined prior to commencement of synthesis; for instance, the type of iron salt used (e.g., iron chlorides and iron bromides), the ratio of Fe^3+^ to Fe^2+^, and the polymer composition are all parameters that are set before synthesis. Studies have shown that altering the iron salt type affects the IONP core size [35,37,50]. This affect is directly correlated to the precursor anion size (Cl^−^, SO_4_^2−^, etc.). The larger these anions are, the more they impede diffusion around the IONP nuclei; therefore, using iron salts with large anions will result in smaller IONPs. IONPs generally have a face-centered cubic structure and because of this, Fe^3+^ and Fe^2+^ atoms reside in different sites in the iron oxide crystal lattice. The availability and probability of these atoms to insert themselves onto the proper lattice sites is the factor that influences the particle size and magnetic properties.

Multiple polymer compositions have been explored for IONP encapsulation including poly-L-lysine, chitosan, dextran, poly(ethylene glycol), and many others. The polymer composition used during synthesis impacts the resulting iron oxide core size of IONPs based on how efficiently the polymer encapsulates the particles. Furthermore, polymer composition influences hydrodynamic size, which is a measure of the IONPs’ core, polymer coating, and the solvent layer that forms in aqueous solution, because different polymers will interact uniquely in solution based on their level of hydrophilicity. Studies have also shown that polymer composition can drastically alter the observed hydrodynamic size and magnetic properties of IONPs [39,42].

Alternatively, parameters such as polymer concentration, temperature, [29] and ratio of iron solution to base solution may be altered and automatically controlled in microfluidic-device-aided coprecipitation synthesis [75]. Increasing the amount of the polymer during coprecipitation synthesis yields smaller IONPs due to the faster rate of particle encapsulation that occurs with more polymer present. The polymer concentration can be altered during coprecipitation synthesis with microfluidics by changing the flow rate of the channel carrying the polymer solution. One study used a coprecipitation process to synthesize IONPs and found that increasing the concentration of polymer greatly decreased particle core size and increased the yield [39].

Iron precursor concentration has also been explored as a potential factor to control IONP core size. Higher concentrations of iron in solution means that more nucleation sites can form. It has been observed that increasing iron concentration decreased overall particle hydrodynamic diameter [67]. This concentration can be controlled by altering the initial solution or by varying the iron solution flow rate, assuming turbulent mixing is not a result. This point highlights a major advantage of these systems in that if a continuous-flow system can be monitored and variation in particle size detected, the flow rate can be altered to compensate for any changes in particle size dynamically. It is also known that increasing base solution concentration decreases particle core size due to the larger drop in pH, which allows more nucleation sites to form. Furthermore, it is known that different types of base solution can also impact particle size independent of the pH experienced in the solution because large base cations hinder the agglomeration of IONPs, resulting in smaller IONP core sizes.

Temperature can be varied to affect particle size as well. Temperature influences the rate of diffusion and in turn how uniformly the particles can grow. Higher temperatures provide the solution with more energy, meaning that the ions in solution can move more vigorously. One study showed increasing the temperature decreased particle core size [29]. It was also observed that increasing the amount of time the IONPs are left at elevated temperatures increased IONP core size [29]. These observations are likely due to aggregation having a large effect on particle size and to aggregation being more strongly affected by residence time than temperature [29]. Residence time is strongly correlated to the flow rate, and as such the IONP core size can be controlled by adjusting flow rates [70]. Interestingly, when heat is removed from a continuous-flow system, there is an ideal flow rate that produces the smallest IONPs [39]. This aspect is seen because there exists an ideal flow rate that maximizes the laminar flow condition of the device.

It is also necessary to compare the standard method of coprecipitation to the microfluidic method of coprecipitation. Reaction volume is the controlling factor here in that at smaller reaction volumes more homogenous solutions are present. It has been shown that both particle size and particle size variation are smaller in microfluidic systems when compared to the standard method [29]. In addition, continuous-flow systems offer a plethora of other parameters, such as presence of gas, that can be implemented and controlled to fine tune the system.

### 5.2. The Effect of Channel Dimensions on Synthesis of IONPs

Channel geometries can have an impact on the microfluidic synthesis of IONPs. The microchannel geometry can affect droplet formation, capillary number, and Reynolds number. Channel geometry can also impact the local mixing experienced by drop-wise flow reactors and can be used to enhance mixing in continuous-flow reactors [42]. Microfluidic channels can also be used to form separate droplets of the iron and base solution, and then using the channels geometry, these separate droplets can be combined into one droplet with the aid of an electric field [42]. This is another attractive use because it can create highly homogenous droplets that are smaller and more monodisperse when compared to IONPs synthesized via the standard method.

The effect of channel height on IONP coprecipitation has also been investigated both experimentally and theoretically in a drop-wise flow reactor synthesizing IONPs [76]. Theoretically, the flow was simulated using a laminar flow model with the Navier-Stokes equation and the equation of continuity, and the produced prediction aligned well with what was experimentally determined [76]. This highlights the ability to evaluate and model microfluidic systems to optimize the system before running any physical experiments. Channels were made at 20, 40, and 60 µm deep. It was determined that a channel of 20 µm deep yielded the smallest core particle sizes and most uniform IONP cores with a size of 4.70 ± 0.90 nm [76]. This fact was attributed to the more efficient mixing of droplets in the channels of smaller heights [76]. In drop-wise flow reactors, increased mixing helps enhance the homogeneity of the solutions. Overall, the channel dimensions have an effect on the ideal mixing time for synthesis of particles [77].

Channel length is one of the main factors determining how long the IONPs are exposed to elevated temperatures. Increasing channel length can cause inconsistencies with pressure and can drive up cost if an expensive carrier solution is being used and recycled. Therefore, it is much more common to alter the flow rates in the microfluidic chip to control the residence times. It has been reported that increasing residence times increases IONP size [29,70]. Namely, a residence time of 37 s produced IONPs with a particle size of 6.3 nm and with a residence time of 75 s IONPs with particle sizes of 9.8 were produced [70]. Particle sizes of 5 nm at a residence time of 150 s and 11 nm at 19 min have also been observed [29]. This affect provides a well-controlled method to fine tune IONP size during synthesis.

### 5.3. Scale-Up Synthesis of IONPs

Large-scale production of IONPs is of paramount importance when commercialization of the product is intended. A major challenge with large-scale production is preserving the attributes of the IONPs such as core size and monodispersity. Another factor that needs to be considered is production rate. This is measured by the mass of IONP that is made in a given amount of time. One study reported synthesizing IONPs with core sizes ranging from 73.3 to 245.5 nm with a production rate of 4.37 g/h [78]. The iron conversion rate was estimated to be 97.6% in the microfluidic synthesis and 96.4% in the standard method [78]. These IONPs were also compared to those produced by the standard method and it was shown that the IONPs synthesized via the microfluidic device were both smaller and more monodisperse than those produced by the standard method IONPs. In comparison, the standard coprecipitation method can generally take up to a few hours to yield a batch of IONPs with production rates of 1.40 g/h [79]. The microfluidic approach has a clear advantage in production rate [78,79].

Unlike the standard approach, the batch size is only limited by the reservoir volumes of the solutions which minimizes set up and shutdown times to further increase efficiency beyond the production rate of a single batch [78,79,80]. Additionally, the production rate of IONPs using microfluidic devices can be further increased over the standard approach by running multiple microfluidic reactors in parallel [78], while better maintaining consistency. This study highlights the microfluidic device’s ability to produce large amounts of IONPs and overcome the batch synthesis method’s limitation on production rate. Furthermore, they showed that they were still able to maintain the monodispersity of produced IONPs at these large production rates [78].

## 6. Conclusions and Outlook

The coprecipitation synthesis of IONPs in microfluidic devices provides a promising alternative to the classical technique of adding a base into a reservoir solution of iron salts. Microfluidic chips allow precise control over the flow rates of the iron solution and base solution as they meet to begin nucleation and growth into IONPs; in turn, the control of fluid flow rates provides a way to fine tune the size of the resulting IONPs. Furthermore, microfluidic devices have been shown to be able to manufacture IONPs with highly reproducible properties between batches due to the high degree of control over the microenvironment where the coprecipitation reaction occurs.

As demand for IONPs increases, a comprehensive and well characterized synthesis technique will be necessary to meet this demand. The effectiveness of microfluidic architectures can be largely attributed to either the Reynolds number, capillary number, or channel friction. Channel accuracy and roughness can also be heavily affected by choice of manufacturing technique. Material choice can determine the availability of manufacturing methods and other reaction parameters such as chemicals used and reaction temperature. Additionally, common standard coprecipitation parameters, such as iron to polymer ratio and polymer type can affect particle size.

Microfluidic devices for synthesis of IONPs can also be expanded to synthesis of other materials by changing the initial solutions. Microfluidic systems have been shown to be able to synthesize nanoparticles with a broad range of materials including polymer [81], cobalt [82], gold [83], and silica [84]. These devices have also demonstrated their versatility in synthesizing nanomaterials of different morphologies including nanospheres [38], nanorods [38], and nanocubes [38]. These materials and structures can also be made at large scales and with short reaction times using microfluidics to enhance the accessibility to these materials.

The choice of substrate materials for fabrication of a microfluidic device is somewhat broad; researchers have successfully employed glass, PDMS, PMMA, silicon, and metals to create microfluidic devices for synthesizing IONPs. Each of these materials has its advantages, disadvantages, and compatible fabrication techniques. Nevertheless, all of the materials mentioned in this review represent an economical and scalable platform for the production of large quantities of highly monodispersed, high-quality IONPs. One of the main advantages to this technique is the ability to continuously produce nanoparticles, eliminating batch-to-batch inconsistencies. However, there could be variations in particle properties during long production times, which can be monitored by simple characterization techniques post synthesis, and then proper alterations can be made to correct the inconsistencies [67]. Additionally, channel fouling is a prevalent issue in many reactors, which can be overcome with channel coatings or changes in channel architecture.

Further work needs to be performed to systematically evaluate IONP synthesis across multiple microfluidic platforms. To better determine the aspects of the microfluidic device design and manufacturing that have the greatest influence on IONP properties, a standard formulation for IONPs should be developed and then tested in microfluidic devices manufactured using different techniques and materials. Additionally, optimization of scale-up systems, particularly in the development of systems that employ multiple chips placed in parallel, needs to be undertaken to provide means to scale up IONP production for clinical use while maintaining key functional properties such as biocompatibility, surface alterability, and magnetic properties. With continued development and testing, microfluidic devices will be well situated to provide a precise, reproducible synthesis method for the large-scale coprecipitation of IONPs.
